# Applying Iterative RE-AIM to Translate Evaluation Data Into Real-Time Adaptations

**DOI:** 10.1093/geroni/igaf122.2065

**Published:** 2025-12-31

**Authors:** Amy Recker, Ashley Smith, Sophia Geisser, Vanessa Aguilar, Amanda Piechota, Rosa Baier, Ellen McCreedy, A Lynn Snow

**Affiliations:** Brown University School of Public Health, Providence, Rhode Island, United States; The University of Alabama, Tuscaloosa, Alabama, United States; University of Alabama, Tuscaloosa, Alabama, United States; The University of Alabama, Tuscaloosa, Alabama, United States; University of Alabama, Tuscaloosa, Alabama, United States; Brown University School of Public Health, Providence, Rhode Island, United States; Brown University, School of Public Health, Providence, Rhode Island, United States; The University of Alabama, Tuscaloosa, Alabama, United States

## Abstract

Iterative RE-AIM provides a novel approach to evaluating interventions in real-time and translating those insights into actionable adaptations. Using the principles of iterative RE-AIM, we conducted a midpoint survey with the members of the coaching intervention team and the implementation evaluation team for 40Winks, a nursing home-based sleep trial. We convened a facilitated meeting with these two teams to discuss results and priorities, evaluate existing adaptations, review the effectiveness of the current intervention plan, propose future adaptations, and formulate a plan for implementation. The aggregated survey data were displayed and discussed by the two teams together as part of this process. While iterative RE-AIM outlines a process for evaluating an intervention’s adherence to the RE-AIM model, we applied this framework to evaluate the intervention’s components rather than the RE-AIM dimensions. This modification allowed us to bring all levels of the intervention and evaluation teams together to have a combined voice in the direction and evaluation of the intervention, and to make critical, real-time adaptations to enhance the effectiveness and reach of the intervention. This presentation will provide a roadmap of the process our team developed, guided by the principles of iterative RE-AIM, to display the evaluation survey and data and discuss strategies for implementing the results of this survey to lead real-time adaptations. This novel methodology allows the teams to learn from and act upon their own data in real time, allowing for iterative and systematic improvements in an intervention as a trial progresses.

